# Children, parents and pets exercising together (CPET): exploratory randomised controlled trial

**DOI:** 10.1186/1471-2458-13-1096

**Published:** 2013-11-27

**Authors:** Ryan Morrison, John J Reilly, Victoria Penpraze, Carri Westgarth, Dianne S Ward, Nanette Mutrie, Pippa Hutchison, David Young, Lindsay McNicol, Michael Calvert, Philippa S Yam

**Affiliations:** 1 School of Veterinary Medicine, College of Medical, Veterinary and Life Sciences, University of Glasgow, Glasgow, Scotland; 2 Physical Activity for Health Group, University of Strathclyde, Glasgow, Scotland; 3 School of Life Sciences, College of Medical, Veterinary and Life Sciences, University of Glasgow, Glasgow, Scotland; 4 Institute of Infection and Global Health, University of Liverpool, Liverpool, England; 5 Gillings School of Global Public Health, University of North Carolina, Chapel Hill, North Carolina, USA; 6 Sport, Physical Education & Health Sciences, University of Edinburgh, Edinburgh, Scotland; 7 Department of Mathematics and Statistics, University of Strathclyde, Glasgow, Scotland

**Keywords:** CPET, Physical activity, Children, Families, Dogs, Accelerometry, Intervention, Feasibility, Acceptability

## Abstract

**Background:**

Levels of physical activity (PA) in UK children are much lower than recommended and novel approaches to its promotion are needed. The Children, Parents and Pets Exercising Together (CPET) study is the first exploratory randomised controlled trial (RCT) to develop and evaluate an intervention aimed at dog-based PA promotion in families. CPET aimed to assess the feasibility, acceptability and potential efficacy of a theory-driven, family-based, dog walking intervention for 9–11 year olds.

**Methods:**

Twenty-eight families were allocated randomly to either receive a 10-week dog based PA intervention or to a control group. Families in the intervention group were motivated and supported to increase the frequency, intensity and duration of dog walking using a number of behaviour change techniques. Parents in the intervention group were asked to complete a short study exit questionnaire. In addition, focus groups with parents and children in the intervention group, and with key stakeholders were undertaken. The primary outcome measure was 10 week change in total volume of PA using the mean accelerometer count per minute (cpm). Intervention and control groups were compared using analysis of covariance. Analysis was performed on an intention to treat basis.

**Results:**

Twenty five families were retained at follow up (89%) and 97% of all outcome data were collected at baseline and follow up. Thirteen of 14 (93%) intervention group parents available at follow up completed the study exit questionnaire and noted that study outcome measures were acceptable. There was a mean difference in child total volume of PA of 27 cpm (95% CI -70, 123) and -3 cpm (95% CI -60, 54) for intervention and control group children, respectively. This was not statistically significant. Approximately 21% of dog walking time for parents and 39% of dog walking time for children was moderate-vigorous PA.

**Conclusions:**

The acceptability of the CPET intervention and outcome measures was high. Using pet dogs as the agent of lifestyle change in PA interventions in children and their parents is both feasible and acceptable, but did not result in a significant increase in child PA *in this exploratory trial*.

**Trial registration:**

ISRCTN85939423

## Background

Levels of objectively measured moderate-vigorous intensity physical activity (MVPA) are much lower than recommended in UK children [1-4], a worrying observation considering that MVPA is associated with numerous health benefits including significant improvements in blood lipids, BMI and body fatness [5]. Recent evidence from the UK suggests that objectively measured PA declines and sedentary behaviour increases before adolescence [4-7]. Furthermore, the incidence of obesity and the degree of excessive weight gain in those who do not become obese is greatest in mid-late childhood (ages 7–11 years old) [8,9]. Recent reviews of interventions to promote PA in children have questioned both the magnitude and sustainability of intervention effects [10-12]. Few family/home based interventions have been carried out to date [13,14], even though some family and home factors are associated with low levels of objectively measured PA in mid-late childhood [13-15]. Therefore, there is an opportunity for novel approaches to the promotion of PA in mid-late childhood incorporating a family/home based intervention.

A study protocol paper has been published detailing the rationale, study design and methods of the Children, Parents and Pets Exercising Together (CPET) exploratory randomised controlled trial (RCT) [16]. Briefly, the use of pet dogs represents a potentially valuable and underutilised opportunity to promote PA in children and their families [17]. In Scotland there are approximately 800,000 dogs and 360,000 children of primary school age, with UK dog ownership estimated at around 22-24% of households with higher dog ownership among families of lower socio-economic status [18]. A number of cross sectional studies have shown that PA levels are higher in adults who walk their dogs regularly [19] and in children who own a dog [20,21]. However, in one Australian study only 23% of 5–6 year olds and 37% of 10–12 year olds ever walked with their dog [22]. Moreover, a recent North American study found that adult dog owners receiving a low intensity dog walking intervention focused on the benefits of walking the dog increased their PA levels more than owners who did not receive the intervention [23]. In summary, promotion of more walking and play with the dog could be a useful strategy to promote family, and in particular, child PA.

There is a dearth of objective information on the type of dog walking by owners and their families; previous estimates of the frequency and duration of dog walking are derived solely from subjective measures such as questionnaires. One recent Australian study found that only 23% of owners walked their dog 5 or more times per week [24] and a study in North America found, that among those who reported walking their dog, the median frequency was 3 times per week with a median duration of 25 minutes [25]. Another Australian study found that children who owned a dog walked the dog on average 1.7 times per week and 32% of owners reported that they rarely or never walked their dog as a family [22]. None of these studies measured dog walking objectively or assessed the intensity of PA achieved during dog walking, an important consideration for maximal health benefits. The present exploratory trial was intended to improve our understanding of the frequency, intensity and duration of dog walking among dog owning families in Scotland.

The UK Medical Research Council Framework for the development and evaluation of complex interventions in public health [26] recommends that definitive trials should be preceded by exploratory trials to provide information about the design, feasibility and acceptability of a trial and intervention. Information regarding the delivery of the intervention, recruitment and retention rates, and effect sizes of outcomes can also be gleaned to help inform future more definitive trials. For example, a recent pilot study exploring the feasibility and acceptability of an intervention aimed at promoting movement skill and PA in children found that recruitment and retention rates, collection of outcome data and delivery of intervention sessions were all high, and staff involved in implementing the intervention reported high satisfaction with the program [27]. In contrast a feasibility study evaluating an adolescent sexual health intervention found that the intervention was unlikely to be deliverable, leading to substantial changes to both the content and delivery of the original intervention [28]. Therefore, an exploratory evaluation of an intervention is required to ensure that subsequent interventions are appropriately designed and developed.

The aim of this paper is to report on the feasibility of the CPET intervention and trial, the acceptability of the trial and intervention, preliminary evidence of its potential efficacy, planning and powering a future intervention, and to improve our understanding of the frequency, intensity and duration of dog walking among dog owning families in Scotland.

## Methods

The following section is a brief account of the study design/methods. For a more detailed description see the study protocol paper [16].

### Design, randomisation, blinding

CPET was an individual, exploratory RCT, and followed guidance on the conduct and reporting of RCT outlined in the CONSORT statement [29]. After baseline outcome measures were made, participating families were allocated randomly to intervention or control group in the ratio of 1.5: 1, respectively, using random number generation in Minitab.

The researcher conducting the outcome measures was blinded to group allocation, and was based at a separate site from the researcher responsible for carrying out the intervention.

### Trial feasibility

#### Study sample, recruitment, and inclusion criteria

**Figure 1:**
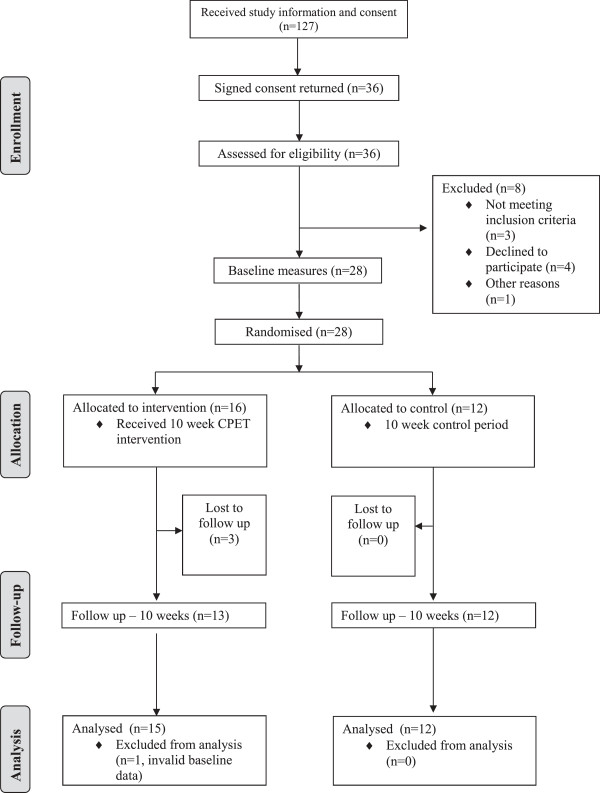
Flow of the study participants.

Invitation letters were sent to approximately 350 dog-owning parents with children attending mainstream primary schools in one local authority area, East Dunbartonshire, in the West of Scotland. Families that responded to the letter were included only if they met the inclusion criteria [16]. Parents were asked to complete a dog behaviour screening questionnaire developed by two members of the Association of Pet Behaviour Counsellors (Westgarth and Hutchison) which assessed whether it was appropriate for each dog to take part in the study. Dogs that did not pass the screening questionnaire and those which were physically unable to take part were excluded. Finally, a CONSORT study flow diagram [29] (Figure 1) was used to summarise sample attrition and missing data for all of the outcome measures. Data on exclusion, recruitment, retention, number of missed intervention sessions and number of completed outcome measures are provided as an indicator of trial feasibility. The Carstairs Score [30] was used as a proxy for socio-economic status of study participants. The Carstairs score is a deprivation measure deriving from UK census data and each postcode area is classified into a deprivation category (DEPCAT for short) ranging from 1 (most affluent) to 7 (most deprived).

### Intervention and control groups

The intervention group participated in a staggered 10-week intervention, from March 2012 to June 2012, which aimed to increase the frequency, intensity, and duration of dog-walking/playing with the family dog. The intervention relied most heavily on modification of the family environment, and the importance of parental support for child PA [10,13-15] was emphasised throughout. The intervention used a number of participant-centred behavioural change techniques including decisional balance, goal setting and relapse prevention [16]. The main focus of the intervention was to target children, parents and the pet dog being physically active together by providing information on dog walking routes and promoting various forms of active play with the dog both indoors and outdoors. Intervention families received one home visit in week 0 (at baseline following outcome measures) from a qualified animal behaviourist and two further home visits in weeks 1 and 6 from a PA research assistant. In addition, intervention families received telephone calls (weeks 2 and 8) and text messages (weeks 4 and 10) to review goal progress, address questions and provide encouragement. The control group did not receive any of the information/content delivered to the intervention group and were asked to carry on as normal for the duration of the study.

### Acceptability of the intervention

As part of the study’s process evaluation and to inform future interventions, a brief study exit questionnaire with Likert scale responses was used to obtain feedback on the intervention from all families in the intervention group, to identify perceived barriers and facilitators of the intervention, perceptions of the acceptability of the intervention and the outcome measures and suggestions for future interventions.

A qualitative study was conducted once the intervention was complete involving a focus group with 6 participating parents from the intervention group, a separate focus group with 6 participating children from the intervention group, and interviews with four key stakeholders (a vet; a pet behaviour counsellor; a walking development officer; and a public health policy manager). A qualitative study of this kind is useful in exploratory studies [31] and in the context of the present study was used to: inform the process evaluation of the intervention; obtain participant views on the acceptability of the intervention and outcome measures; obtain participant suggestions for the intervention to be developed for a future larger, longer term, trial.

### Baseline dog walking

Parents were asked about the frequency and duration of dog walking. Dog walking was also identified by analysis of simultaneous accelerometer records, both parent only dog walking (assessment of parent and dog accelerometer records) and family dog walking (assessment of parent, child and dog accelerometer records) at baseline.

### Potential efficacy

#### Outcome measures

Outcomes were measured at baseline and 11 weeks later (in the week after the end of the intervention for the intervention group). The primary outcome was 10 week change (baseline-1 week post-intervention) in objectively measured total volume of PA with the Actigraph accelerometer (ActiGraph, Pensacola Fl) using the accelerometry counts per minute (cpm) averaged over 7 days in the children. Secondary outcomes in children were changes in objectively measured light intensity PA (800–3200 cpm [32]), MVPA (≥3200 cpm [32]) and sedentary behaviour (<800 cpm [32]) using the validated cut points of Puyau et al. [32], and changes in sitting time using the pragmatic cut-point, not yet validated and calibrated, of 150 cpm [33]. PA and sedentary behaviour in children were measured using the Actigraph GT3X + accelerometer (The Actigraph, Florida). Further secondary outcomes in children were: changes in body composition (fat mass index and lean mass index) and in whole body and lumbar spine bone mineral content using a Lunar Prodigy whole-body dual energy X-ray absorptiometry (DXA) scanner (GE Medical Systems, Madison, WI) in conjunction with encore software version 13; changes in body weight and in BMI z scores expressed relative to UK 1990 reference data; and changes in Child Health Related Quality of Life, as reported separately by both the children and their parents, using the PedsQL which is practical, valid and sensitive to change resulting from lifestyle interventions [34,35].

Secondary outcomes in parents were: changes in objectively measured total volume of PA, over 7 days, with the Actigraph GT1M accelerometer (The Actigraph, Florida) using the accelerometry cpm; changes in light intensity PA (100–1951 cpm [36]), MVPA(≥ 1952 cpm [36]) and sedentary behaviour (<100 cpm [36]) using the validated cut points of Freedson et al. [36]; and changes in body weight and BMI. Secondary outcomes in dogs were changes in objectively measured total volume of PA, over 7 days, with the Actigraph GT3X + accelerometer (The Actigraph, Florida); changes in light-moderate intensity PA (1352–5695 cpm [37]), vigorous intensity PA (≥5696 cpm [37]) and sedentary behaviour (<1352 cpm [37]) using the validated cut points of Morrison et al. [37]; and changes in body weight and body condition score [38].

Finally we measured changes in family dog walking behaviour (number of walks/week; total duration of walking time; average duration of walking time; child mean accelerometer cpm; percent time child spent in MVPA; parent mean accelerometer cpm; percent time parent spent in MVPA; dog mean accelerometer cpm; and percent time dog spent in vigorous intensity PA) by assessing simultaneous Actigraph accelerometry data from parent, child, and dog, identifying periods when all three were physically active together. Valid accelerometry data was defined as a minimum of 3 consecutive days and a minimum of 6 hours per day in children [39], a minimum of 3 consecutive days [40] and a minimum of 10 hours per day in adults [41], and a minimum of 3 consecutive days in dogs [42]. Participants were asked to record periods when the accelerometer was not worn in an activity diary.

#### Statistical analysis and sample size considerations

A pre-study power calculation suggested that it would be possible to detect an increase in daily accelerometry counts in the intervention group equivalent to 187 cpm with no change in the control group, with around 15 families per group [16]. Statistical analysis was carried out on both an intention to treat and per protocol basis. For the intention to treat analysis missing data were replaced using the last measure carried forward method. Any family with missing child, parent or dog accelerometry data were excluded from analysis of family dog walking behaviour. The intervention and control groups were compared using analysis of covariance, with the follow-up measure as the dependent variable, group as the independent variable, and the baseline measure as the covariate [43]. All analyses were carried out using Minitab 16.1.1.

### Planning and powering future interventions

CPET is an exploratory study, with the main focus to report on the feasibility and acceptability of the intervention; however CPET was also intended in part to inform future sample size calculations for the outcome measures used. A post-hoc power calculation was carried out using Minitab 16.1.1 to estimate the sample size needed to detect differences in accelerometer cpm in a future, larger scale trial.

### Ethical and safety considerations

The study was approved by the University of Glasgow College of Medical, Veterinary, and Life Sciences Ethics Committee, the University of Glasgow School of Veterinary Medicine Ethics and Welfare Committee, and the University of Liverpool Leahurst Research Ethics Committee. Informed written consent to participation was received from each participating child, and from each participating parent.

Safety of the intervention was enhanced by emphasising that children should not walk the dog alone, but along with the parent.

## Results

### Feasibility of the trial

Of the 37 primary schools approached, all but 2 gave permission for researchers to discuss with and give information to children about the study. One-hundred and twenty-seven dog owning families returned a note of interest in the study and were subsequently sent information packs and consent forms. Of those 127 families, 36 (28%) returned signed consent forms. Four (11%) of the 36 families who returned consent subsequently declined to participate, 3 (8%) did not meet our inclusion criteria (1 family was excluded on the grounds of their dog being too aggressive and the other 2 due to medical conditions with their dog), and 1 family was non-contactable (3%).

Twenty-eight families (22% of those who expressed an interest), two of which had two eligible children, were recruited in the study and 25 (89%) were retained at follow up. One family became disengaged very early on in the intervention and withdrew, another family had to withdraw due to medical reasons and the final family did not respond to telephone calls and it was not possible to arrange follow up measures. Body weight, body mass index (BMI), body condition score (BCS) and child health related quality of life data were collected for all 28 families (100%) at baseline and all 25 families (100%) at follow up. There was only a small number of available DXA appointments after school hours and suitable appointments for some families were unavailable, therefore body composition (lean mass index and fat mass index) and bone mineral content data were collected for 25/30 children (82%) at baseline and for 23/27 children (84%) at follow up. Objectively measured PA data were collected for 30/30 children (100%), 27/28 parents (96%) and 27/28 dogs (96%) at baseline, and 27/27 children (100%), 24/25 parents (96%) and 24/25 dogs at follow up. At baseline, 1 parent wore the accelerometer for less than 2 days and one accelerometer being worn by a dog malfunctioned, therefore these accelerometry data could not be included in the analysis (these participants were from the same family who later withdrew due to disengagement with the intervention). Immediately after baseline measures were carried out, all 15 intervention families were available for the first intervention session delivered by the animal behaviourist. All 15 intervention families were available for the first visit by the PA research assistant, and 14/15 were available for the second visit. Thirteen of 15 intervention families were available for all home visits and telephone calls/text messages, one family missed only one text message and another missed one telephone call and two text messages. Overall 85/90 PA sessions were delivered, with all families who were available at follow up (12) attending all sessions, meaning that 12/15 (80%) families were engaged in the intervention.

### Characteristics of participants

Twenty-eight families were recruited to the study, two of these families had two eligible children, therefore 30 children, 28 parents and 28 dogs were included in the study sample. The flow of study participants is shown in Figure 1. The mean age of children in the sample was 10.9 years with 80% (24/30) classified as healthy weight, 13% (4/30) as overweight and 7% (2/30) as obese. The mean age of parents in the sample was 44.8 years with 50% (14/28) classified as healthy weight, 36% (10/28) as overweight and 14% (4/28) as obese. The mean age of dogs in the sample was 3.7 years with 89% (25/28) classified as healthy weight, 11% (3/28) as overweight and 0% as obese. Sixty-seven percent (20/30) of children and 82% (23/28) of parents who participated were female. Thirty-nine percent (11/28) of families were classified as DEPCAT 1, 39% (11/28) were classified as DEPCAT 2, 18% (5/28) were classified as DEPCAT 3, and the remaining 4% (1/28) classified as DEPCAT 6.

### Baseline PA and dog walking

Mean total volume of PA in children was 522 (SD 125) cpm at baseline. Children spent 80% (SD 4) of their waking time sedentary (equivalent to > 9 hours/d), 16% (SD 3) in light intensity PA (equivalent to ~ 1 hour 53 mins/d), and 3% (SD 2) in MVPA (equivalent to ~ 21 mins/d). The mean length of sitting bouts in children was 5 (SD 1) minutes, with a mean of 12 (SD 2) breaks per sitting hour and children spent 14% (SD 6) of wear time sitting in bouts longer than 30 minutes. Mean total volume of PA in parents was 430 (SD 148) cpm at baseline.

Parents spent 59% (SD 8) of their waking time sedentary (equivalent to > 8 hours/d), 35% (SD 7) in light intensity PA (equivalent to > 4 hours/d), and 6% (SD 3) in MVPA (equivalent to ~ 50 mins/d). The mean length of sedentary bouts in parents was 6 (SD 2) minutes, with a mean of 10 (SD 2) breaks per sedentary hour and parents spent 17% (SD 8) of wear time in sedentary bouts longer than 30 minutes. Mean total volume of PA in dogs was 818 (SD 230) cpm at baseline. Dogs spent 80% (SD 5) of their waking time sedentary (equivalent to > 13 hours), 17% (SD 4) in light-moderate intensity PA (equivalent to > 2 hours), and 3% (SD 1) in vigorous intensity PA (equivalent to ~ 30 mins/d).

At baseline parents reported that they walked their dog 2–3 times daily with a mean duration of 34 minutes (SD 23) per walk. However, analysis of simultaneous parent and dog (parent only dog walking) accelerometry data suggests parents walked their dog 1–2 times per day with a total duration of 20 (SD 13) minutes per day. When analysis of simultaneous accelerometry data were extended to include children (i.e., family dog walking) dog walking decreased further to an average of less than 1 family walk per day with a total duration of 6 (SD 7) minutes per day. At baseline children spent 21% (SD 19) of dog walking time in MVPA and parents spent 39% (SD 38) in MVPA.

### Acceptability of the intervention

All parents in the intervention group were asked to complete a short study exit questionnaire which was completed by 13/14 (93%) intervention parents who were available at follow up. Eleven of 13 parents (85%) agreed that the intervention content delivered by the animal behaviourist was sufficient to allow them to participate in a safe manner; no parents disagreed. Furthermore, 12/13 (92%) parents agreed that the PA aspects of the intervention content were sufficient to raise awareness of current PA levels. Moreover, 10/13 (77%) of parents agreed that the intervention content was sufficient to motivate them to increase the amount of dog walking they did previously, and 12/13 (92%) agreed that it was sufficient to increase the amount of dog walking that their children did. Parents were also asked about barriers to participation in CPET (lack of motivation, lack of enjoyment, injury/illness, lack of time etc.); 7/13 (54%) parents agreed that they were restricted from taking part due to a lack of time. Finally, parents were asked for their thoughts on the CPET outcome measures. Only 1/13 parents (8%) reported that there were too many outcome measures and none reported that they were asked to do too much PA during the intervention.

Small focus groups were carried out, one with children in the intervention group (n = 6) and the other with parents in the intervention group (n = 6). Both children and parents reported that the acceptability of the intervention and outcome measures was high. Children enjoyed taking part in the CPET study and felt that it made them more active because of the fun games that they played outside and they enjoyed filling in the reward chart. They highlighted that it was a way to decrease screen time and that they bonded more with their dog. Overall, the children reported that CPET “makes you healthy, happy and is lots of fun”. Parents felt that the reward chart and knowledge of their baseline PA levels were the catalysts in motivating them to adhere to the 10 week intervention. They reported that they had experienced longer, more fun family walks. Some parents reported that their children were more physically active whilst others felt it had replaced other forms of PA, e.g. playing with friends outdoors. All parents mentioned that the behaviour of their dog was better and they were “happier knowing that my dog and my child were being more active”. Finally, four key stakeholders were interviewed, with all four in agreement that the CPET intervention had great potential to improve health for children, their parents and pet dogs. Challenges highlighted by these stakeholders were around how to motivate families to take part, particularly those from less affluent areas where their immediate environment may not feel safe or attractive for families to explore. Stakeholders repeatedly highlighted practical time issues and when to fit in bouts of dog walking as barriers, particularly for working parents.

### Potential efficacy

The results of the intention to treat analyses are shown in Tables 1 and 2. Change in family dog walking behaviour, sedentary behaviour, total volume of PA and time spent in different intensities of PA are shown. Changes in all other outcomes are shown in Additional file 1, none of which were statistically significant.

**Table 1:** Objectively measured family dog walking behaviour for intervention and control groups

	**Baseline**		**Follow-up**				
	**Intervention mean (SD)**	**Control mean (SD)**	**Intervention mean (SD)**	**Control mean (SD)**	**Intervention –control difference (95% CI)**	**p value‡**	**Effect size (Cohen’s d)**
	**N = 15**	**N = 12**	**N = 15**	**N = 12**			
Number of walks per week	2.7 (2.1)	2.6 (2.6)	2.6 (1.2)	2 (1.7)	0.5 (-0.4, 1.4)	0.19	0.23
Total duration of dog walking (mins/week)	53 (58)	25 (29)	47 (37)	23 (23)	-4 (-25, 17)	0.12	0.10
Child mean accelerometer cpm* during dog walking	2117 (1289)	1953 (1136)	2784 (1279)	2490 (1724)	130 (-539, 799)	0.41	0.10
% time child spent walk in MVPA†	22.1 (21.4)	18.9 (18.6)	26.1 (21.7)	15.2 (21.7)	7.7 (-1.7, 17.1)	0.12	0.32
Parent mean accelerometer cpm* during dog walking	1996 (1673)	1518 (1205)	2216 (1237)	1601 (976)	137 (-486, 760)	0.39	0.10
% time parent spent walk in MVPA§	45.3 (41.2)	31.7 (34.5)	42.0 (30.8)	20.3 (23.8)	8.1 (-6.4, 22.6)	0.11	0.22
Dog mean accelerometer cpm* during dog walking	3595 (2031)	3549 (2572)	4558 (1746)	3766 (2155)	746 (-345, 1837)	0.39	0.27
% time dog spent in light-mod PA¶	60.4 (34.5)	69.4 (39.3)	55.2 (32.4)	37.6 (40.1)	26.6 (6.8, 46.4)	0.06	0.53
% time dog spent in vigorous PA**	19.6 (17.7)	13.9 (23.2)	24.7 (17.3)	12.7 (19.6)	6.3 (-4.2, 16.8)	0.03	0.24

*cpm = counts per minute.

†≥3200 accelerometer cpm.

§≥1952 accelerometer cpm.

¶1352–5965 accelerometer cpm.

**≥5696 accelerometer cpm.

‡Intervention and control groups were compared using analysis of covariance.

Of the 28 families recruited, 1 parent and 1 dog (from the same family) returned invalid accelerometry data at baseline and were excluded from analysis.

**Table 2:** Objectively measured habitual PA and sedentary behaviour for intervention and control groups

	**Baseline**		**Follow-up**		**10-week differences**		
	**Intervention mean (SD)**	**Control mean (SD)**	**Intervention mean (SD)**	**Control mean (SD)**	**Difference in change between intervention and control (95% CI)**	P value**	**Effect size (Cohen’s d)**
**Children**	**N = 17**	**N = 13**	**N = 17**	**N = 13**			
Total volume (mean cpm)*	521 (112)	524 (144)	548 (216)	521 (147)	30 (-23, 82)	0.62	0.21
% time being sedentary†	80.9 (3.4)	80.2 (4.9)	80.7 (5.0)	80.1 (5.3)	-0.1 (-1.8, 2.0)	0.90	0.02
% time in light PA†	16.0 (2.9)	16.7 (3.8)	15.9 (3.6)	16.5 (4.3)	0.1 (-1.5, 1.7)	0.84	0.02
% time in MVPA†	3.1 (1.6)	3.1 (1.9)	3.3 (2.7)	3.0 (1.3)	0.3 (-0.3, 0.9)	0.60	0.20
% time sitting†	56.5 (5.4)	57.0 (7.3)	57.3 (7.7)	56.5 (9.1)	1.3 (-1.6, 4.3)	0.70	0.17
Length of sitting bouts (minutes)	5 (0.5)	6 (1.4)	6 (4.9)	6 (2.1)	1.0 (-0.2. 2.2)	0.66	0.30
Breaks per sitting hour	12.5 (1.4)	12.1 (3.3)	12.0 (2.9)	12.5 (2.9)	-0.1 (-1.3, 1.1)	0.60	0.03
% of wear time in bouts > 30 minutes	12.3 (4.9)	15.8 (7.6)	15.2 (15.7)	17.4 (9.7)	1.3 (-3.0, 5.7)	0.90	0.11
**Parents**	**N = 15**	**N = 12**	**N = 15**	**N = 12**			
Total volume (mean cpm)*	470 (156)	380 (126)	447 (154)	394 (113)	-37 (-62, -13)	0.30	0.60
% time being sedentary§	59.9 (7.7)	58.7 (8.8)	61.0 (7.2)	58.1 (6.70)	2.2 (0., 4.3)	0.18	0.42
% time in light PA§	33.7 (6.8)	36.3 (7.3)	32.2 (6.3)	36.6 (5.3)	-1.9 (-4.0, 0.2)	0.10	0.35
% time in MVPA§	6.9 (3.0)	5.0 (2.9)	6.9 (3.2)	5.3 (2.4)	-0.3 (-1.0, 0.4)	0.96	0.19
Length of sedentary bouts (minutes)	6 (1.2)	6 (2.0)	7 (1.5)	6 (1.6)	1.0 (0.3, 1.2)	0.11	0.60
Breaks per sedentary hour	10.3 (1.8)	10.9 (2.6)	10.0 (1.9)	11.2 (2.2)	0.6 (-0.1, 1.3)	0.20	0.33
% of wear time in outs > 30 minutes	16.2 (6.)	17.6 (9.7)	19.3 (8.9)	16.3 (6.7)	4.4 (1.3, 7.5)	0.18	0.56
**Dogs**	**N = 15**	**N = 12**	**N = 15**	**N = 12**			
Total volume (mean cpm)*	606 (169)	636 (208)	631 (217)	582 (147)	79 (35, 123)	0.09	0.71
% time being sedentary¶	85.8 (3.6)	85.6 (4.3)	84.9 (5.2)	85.9 (3.6)	-1.2 (-2.3, -0.1)	0.31	0.45
% time in light-moderate PA¶	12.5 (3.2)	12.7 (3.7)	13.4 (4.3)	12.8 (3.1)	0.9 (-0.2, 1.9)	0.47	0.34
% time in vigorous PA¶	1.74 (1.0)	1.7 (1.0)	1.8 (1.3)	1.4 (0.8)	0.4 (0.2, 0.6)	0.08	0.70

*cpm = count per minute.

†Sedentary <800 accelerometer cpm; light PA 800–3200 accelerometer cpm; MVPA ≥3200 accelerometer cpm; sitting <150 accelerometer cpm.

§Sedentary <100 accelerometer cpm; light PA 100–1951 accelerometer cpm; MVPA ≥1952 accelerometer cpm.

¶Sedentary <1352 accelerometer cpm; light-moderate PA 1352–5695 accelerometer cpm; vigorous PA ≥5696 accelerometer cpm.

**Intervention and control groups were compared using analysis of covariance.

Mean duration of accelerometry monitoring at baseline in children was 6.8 days (SD 0.6) with mean duration of 12.2 h (SD 1.6) per day. At follow up mean duration of accelerometry monitoring in children was 6.0 days (SD 1.1) with mean duration of 12.3 h (SD 1.8) per day. Mean duration of accelerometry monitoring in parents at baseline was 6.7 days (SD 0.9) with mean duration of 14.3 h (SD 1.3) per day. At follow up mean duration of accelerometry monitoring in parents was 6.5 days (SD 1.2) with mean duration of 14.0 h (SD 2.0) per day. Mean duration of accelerometry monitoring in dogs at baseline was 6.8 days (SD 0.9) with mean duration of 16.8 h (SD 0.5) per day. At follow up mean duration of accelerometry monitoring in dogs was 6.9 days (SD 0.9) with mean duration of 16.6 h (SD 1.0) per day.

Of the 28 families recruited, 1 parent and 1 dog (from the same family) returned invalid accelerometry data at baseline and were excluded from analysis.

#### Change in family dog walking behaviour

Changes in family dog walking behaviour for intervention and control groups are shown in Table 1. There were no significant differences in the number of walks per week or total duration of walks per week. There were also no significant changes in mean accelerometer cpm for children or parents during dog walking, or the amount of time spent in MVPA. There was, however, a significant difference in the amount of time spent in vigorous intensity PA for dogs during dog walks (p = 0.03), although the reported effect size was small (d = 0.24). Effect sizes were also small for all other outcomes except the amount of time dogs spent in light-moderate intensity PA (d = 0.53).

#### Sedentary behaviour and PA

Table 2 shows the results of PA related outcomes. There were no significant differences in the total volume of PA, amount of time being sedentary or the amount of time in light intensity PA or MVPA for children or their parents. Small effect sizes were reported for all PA outcomes in children and parents except for total volume of PA in parents (d = 0.60). The reported effect, however, is negative, i.e., control group parents increased PA from baseline to follow up whereas PA decreased in intervention group parents. Again there were no significant differences in any of the PA outcomes for dogs, however medium to large effect sizes were reported for total volume of PA (d = 0.71) and amount of time spent in vigorous intensity PA (d = 0.70).

### Per protocol analysis

When participants without both baseline and follow up data were removed from the per protocol analyses, results of the statistical significance tests and calculation of effect sizes did not change (data not shown).

### Post-hoc power calculation

A post-hoc power calculation is possible using the mean difference of change in total volume of PA in children (the primary outcome measure) of 30 accelerometer cpm between intervention and control groups, and the square root of the mean squared error as an estimate of standard deviation, 156 accelerometer cpm. With 80% power at p = 0.05, this difference would be detectable with 209 families in each group (intervention and control).

## Discussion

To our knowledge this is the first RCT to assess the feasibility, acceptability and potential efficacy of a dog-based PA intervention in children and their families. CPET was an exploratory, assessor-blinded RCT as recommended in the UK MRC framework for developing and evaluating complex interventions [26], and was developed to inform a future more ‘definitive’ trial. The results show that the CPET trial was feasible and the intervention was acceptable to participants. Eighty-nine per cent of families were retained in the present study and greater than 90% of data were collected for all outcome measures except those deriving from DXA scans. Children and parents who participated in the intervention group agreed that the outcome measures and intervention content were acceptable. Despite this, there was no significant change in the primary outcome measure (child PA) or the majority of the secondary measures (parent and dog PA, child BMI, bone health and health related quality of life). Furthermore, small effect sizes were found for our primary outcome measure and all secondary outcome measures except total volume of PA in dogs and the amount of time they spent in vigorous intensity PA.

The retention rates in CPET are comparable to those reported in other PA studies in children and their families. Chen et al. [44] retained 85% of participants at follow up and Sacher et al. [45] reported that 90% of children randomised to a 9 week intervention were available at the end of the intervention. Few family based studies have reported the level of adherence to the intervention programme. Morgan et al. [46] reported that 81% of intervention sessions were attended and Sacher et al. [45] reported that 86% of sessions were attended during the intervention. The level of adherence to the CPET intervention programme and the results from our qualitative study suggest that acceptability of the outcome measures and intervention content was high. One possible explanation for the high rate of adherence to the intervention in CPET may be that all intervention sessions were home/telephone based whereas other intervention programmes have required attendance at group sessions outside the home [45,46]. The CPET intervention was therefore flexible in terms of delivery, participants were not required to travel in order to receive the intervention and all intervention sessions were delivered to suit the availability of participants.

The number of completed outcome measures in CPET was high for all outcomes except measurement of fat mass, lean mass and bone mineral content in children. Body weight, BMI, BCS and child health related quality of life data were collected for all participants at baseline and all available participants at follow up, indicating that feasibility of collecting these outcomes was high. Furthermore objectively measured PA data were collected on all children at baseline and all those available at follow up. PA data was collected on all but one parent and dog at baseline, and all but one parent and dog that were available at follow up. One reason why only 82% of DXA related outcome data was collected at baseline and from 84% of the available children at follow up was because participants were asked to attend a local children’s hospital to have DXA scans carried out but only limited appointments were after school hours. Some participants were therefore unable to make a suitable appointment due to other commitments.

Although a dog-walking intervention such as CPET may be both feasible and acceptable on a small scale and in the short term, the relatively low conversion of expressions of interest (n = 127) into signed consent (n = 36) suggests that alternative methods of recruitment may be required to implement CPET on a larger scale. Prior to a definitive trial a formative evaluation of the reasons why those who initially expressed an interest and subsequently did not choose to take part in the study might highlight reasons for the low conversion rate. It may be that the expansion of CPET will require recruitment to be carried out over a larger area with access to more potential participants. Alternatively a completely different approach to recruitment such as advertising in local media or veterinary practices may be necessary. The primary concern with regards to recruitment was a potentially high rate of exclusion due to behavioural issues with dogs, however this did not seem to be the case as only 1 of the 36 families who consented to participation was excluded for this reason.

No statistically significant differences in either the number or duration of dog walks, or child or parent PA during periods of dog walking were detected and effect sizes were small for all outcomes. However, a statistically significant difference in the amount of time dogs spent in vigorous intensity PA during dog walks was found. It should be noted that although this was statistically significant, it equates to a mean difference of ~ 2 minutes during total dog walking time for the week, and is therefore unlikely to be of any biological significance. If the number of dog walks in intervention families had increased however, the amount of time dogs spent in vigorous intensity PA may have been much greater. Previous reports on the amount of family dog walking have relied on subjective measures such as questionnaires [22], which are often subject to response bias [47,48]. The present study presents data not only on the amount but the intensity at which dog walking takes place among families and suggests that dogs are walked 2–3 times per week as a family when measured objectively. This suggests that in our sample family dog walking was more common than elsewhere, with one Australian study reporting that when measured subjectively family dog walking took place at most once or twice per month [22]. Nevertheless, the amount of family dog walking in this study was still low considering that the average total duration per week for the sample as a whole was 41 minutes at baseline and 37 minutes at follow up. Despite no significant increase in PA being observed due to the intervention, the extent to which dog walking might contribute to accumulated MVPA in children and/or their parents suggests that increasing the frequency and duration of dog walking is a promising strategy for increasing PA in families. Children spent between 15 and 26% of family dog walking time in MVPA compared to only 3% of total wear time; parents spent between 20 and 45% of dog walking time in MVPA compared to between 5 and 7% of total wear time; and dogs spent 13-25% of dog walking time in vigorous intensity PA compared to only 1-2% of total wear time. This suggests that, if carried out for a sufficient duration, family dog walking represents an opportunity to increase MVPA across the whole family and might in future be valuable for PA promotion more widely. The amount of time spent sedentary or physically active can vary widely depending on the cut point used to define intensities of PA in both children and adults [49], it is therefore difficult to say with certainty that periods when the parent, child and dog were all being physically active together were correctly identified.

Although effect sizes were small for our primary outcome measure (total volume of PA in children) they were not too dissimilar to many other PA interventions in children. A recent meta-analysis [50] which assessed the effectiveness of 30 PA interventions where PA was measured objectively (i.e., accelerometry) concluded that such interventions have had a small to negligible effect on total volume of PA (d = 0.12) and with only small increases in MVPA, ~ 4 minutes per day (d = 0.16). The CPET intervention resulted in a mean difference in total volume of PA of 30 cpm in children (d = 0.21) and a mean difference in MVPA of ~ 2 mins/day (d = 0.20), although given the lack of power in the present study it is questionable whether these differences are real. In contrast the effect sizes reported here for PA in parents did not compare favourably with other PA interventions in adults, with a recent review [51] concluding that the pooled intervention effect on overall PA between intervention and control groups was small (d = 0.19). The results for the efficacy of the intervention on dogs were more positive, with medium to large effect sizes reported for total volume of PA (mean difference of 79 cpm, d = 0.71) and vigorous intensity PA (mean difference of ~ 6 minutes/day, d = 0.70). To our knowledge there are no published studies assessing the efficacy of a PA intervention on objectively measured PA levels of pet dogs and therefore comparison with other studies is not possible. Furthermore, the biological significance of an increase in vigorous intensity behaviour of 6 minutes/day in dogs is unclear.

There are no comparable studies assessing the efficacy of a dog walking intervention on PA in children, although there are two published studies assessing the efficacy of a dog walking programme in adults. Kushner et al. [52] reported that overweight adults taking part in a 16 week intervention with pet dogs had significant increases in PA and significant decreases in body weight. PA was not measured objectively and this was a clinical trial in overweight adults, and it is therefore difficult to make comparisons with CPET. Rhodes et al. [23] carried out a pilot study of a dog walking intervention in adults who did not regularly walk their dog: their intervention appears to have resulted in significantly higher step counts compared to control. In contrast CPET did not result in an increase in objectively measured PA in either children or their parents, or the number or duration of dog walks. In the present study children and their parents were asked to participate in dog walking/active play with the dog together and this may be logistically more difficult than asking a lone adult to increase dog walking time and thus PA [23]. In addition, dogs were already being walked at baseline and a number of parents taking part in CPET noted at baseline that it was difficult for them to change their habits of dog walking. These parents liked to walk the dog early in the morning or late at night, i.e. when their child would be less likely to take part. Future dog walking interventions should attempt to help families reduce barriers (e.g., time) and create more opportunities for additional dog-walking opportunities as a family.

Despite the overall lack of effect that CPET had on PA outcomes the results of the qualitative study were promising. The vast majority of parents in the intervention group agreed that the intervention content was sufficient to motivate them and their children to increase PA through dog walking. In addition, children who took part in the focus groups highlighted that they enjoyed taking part in CPET and felt that they increased the amount of PA they did. Similarly parents who took part in the focus groups suggested that they had experienced longer family walks. Key stakeholders interviewed as part of the qualitative study suggested that it may be difficult to motivate families from less affluent areas to take part. The distribution of DEPCAT scores among the study population suggests that this was not an issue in this study. Ninety-six percent (27/28) of CPET families were classified as DEPCAT 1, 2 or 3 compared to 87% of the population of East Dunbartonshire and 42% of the population of Scotland [30]. This all suggests that motivation was not a factor in the lack of change in PA.

It is possible that the short duration of the intervention prevented an increase in PA in the intervention group compared to controls and a future more definitive trial should include an intervention period of longer than 10 weeks. It may also be possible that intervention families did increase PA during the intervention as suggested in the focus groups but failed to maintain this when PA was measured post intervention. Future trials should, if possible, measure PA multiple times, i.e. at baseline, mid-intervention and post intervention, for longer periods i.e. 2 weeks instead of 1, or even continuously throughout the intervention period to overcome this, although the increased burden of wearing accelerometers for longer periods may not be acceptable to participants. Continuous or multiple measurement of PA would also help overcome any effect of variations in weather. Clearly adverse weather conditions can have an effect on outdoor PA and data from the nearest UK Met Office weather station to the geographical area where the intervention took place estimates that rainfall in the period when many of the post intervention measurements were taken was almost double that at baseline [53]. The failure to detect any significant changes in child or parent PA may also have been due to PA compensation, the theory that study participants may compensate for imposed bouts of PA by reducing PA at other times [54,55]. This was highlighted by some of the parents who took part in the focus groups who suggested that CPET had replaced other forms of PA. Future studies should therefore highlight the importance of maintaining other forms of PA while also increasing the amount of dog walking.

This study had a number of strengths and limitations. First, it was an exploratory randomised controlled trial as set out in the CONSORT statement [29] and the UK Medical Research Council Framework for the development and evaluation of complex interventions in public health [26]. Second, trained blinded assessors were used to carry out the outcome measures and were independent of the research assistant who delivered the intervention. Furthermore, the study methods included validated instruments for measuring our primary and secondary outcomes, including the objective measurement of PA. Although it was not one of our aims to recruit a large sample size, the relatively small sample size prevented assessment of any effects of the intervention on boys and girls separately, or by type of dog owned (breed, age etc.) and is symptomatic of an exploratory trial. The small sample size also prevented conclusions with any certainty as to whether any of the small differences found are real. In addition, the intervention at this stage was resource intense to deliver (for example using a session with a qualified behaviourist). This study was designed as an exploratory RCT that will inform the design of a future, larger and longer term trial, not to report solely on the potential efficacy of such a study. Studies of this nature are needed to determine if dog based PA interventions have any effect on habitual PA over the longer term. Since outcomes were measured in the period immediately after the intervention rather than during the final week this might have reduced any apparent impact of the intervention.

## Conclusions

This study suggests that using pet dogs as the agent of lifestyle change in PA interventions in children and their parents is both feasible and acceptable. These results will be used to inform the design, development and implementation of future, larger scale trials.

## Competing interests

The authors declare that they have no competing interests.

## Authors’ contributions

RM contributed to the design of the study, collected outcome measure data, performed the statistical analysis, and drafted the manuscript. JJR conceived the study and contributed to its design and coordination and helped draft the manuscript. VP contributed to the design of the study, delivered the physical activity content of the intervention and helped draft the manuscript. CW contributed to the design of the study, analysis of dog screening questionnaires and revision of the draft manuscript. DSW contributed to the design of the study and revision of the draft manuscript. NM contributed to the design of the study and revision of the draft manuscript. PH contributed to the design of the study, analysis of dog screening questionnaires and revision of the draft manuscript, and delivered the dog behaviour aspects of the intervention. DY contributed to the design of the study and revision of the draft manuscript. LM and MC contributed to the collection of outcome measure data and revision of the draft manuscript. PSY contributed to the study design and its coordination and helped draft the manuscript. All authors read and approved the final manuscript.

## Pre-publication history

The pre-publication history for this paper can be accessed here:

http://www.biomedcentral.com/1471-2458/13/1096/prepub

## Supplementary Material

Additional file 1 This file includes tables showing data from all outcome measures not shown in the manuscript. Click here for file
