# Supplementary material for: Children, parents and pets exercising together (CPET): exploratory randomised controlled trial
Source: BMC Public Health. 2013 Nov 27;13:1096. doi: 10.1186/1471-2458-13-1096 (PMC4222564; doi:10.1186/1471-2458-13-1096)
Supplement: Additional file 1 — This file includes tables showing data from all outcome measures not shown in the manuscript. [file 1471-2458-13-1096-S1.pdf]

Children, parents and pets exercising together (CPET): exploratory randomised controlled trial.

Supplementary information

Table 3. Body composition related outcome measures for intervention and control groups

	Baseline		Follow-up				
	**Intervention Mean (SD)**	**Control Mean (SD)**	**Intervention Mean (SD)**	**Control Mean (SD)**	**Difference in change between intervention and control (95% CI)**	**P value**	**Effect size (Cohen's d)**
**Children**	**N = 17**	**N = 13**	**N = 17**	**N = 13**			
Body weight (kg)	37.3 (6.1)	39.8 (11.9)	38.3 (6.7)	40.6 (11.8)	0.2 (-0.3, 0.8)	0.69	0.14
BMI (kg/m^2)	18.4 (2.2)	18.7 (4.7)	18.5 (2.3)	18.7 (4.6)	0.1 (-0.1, 0.3)	0.71	0.15
BMI z-score	0.32 (0.90)	0.11 (1.65)	0.28 (0.90)	0.06 (1.51)	0.01 (-0.08, 0.10)	0.77	0.04
	**N = 14**	**N = 11**	**N = 14**	**N = 11**			
Fat Mass Index (kg/m^2)	5.0 (1.9)	5.5 (3.8)	5.1 (2.0)	5.4 (3.8)	0.2 (0.0, 0.4)	0.33	0.39
Lean Mass Index (kg/m^2)	12.2 (0.9)	12.4 (1.3)	13.3 (4.2)	12.5 (1.3)	1.1 (0.2, 1.9)	0.38	0.51
Whole body BMC (g/cm^2)	0.90 (0.04)	0.93 (0.08)	0.89 (0.07)	0.94 (0.08)	-0.02 (-0.04, -0.01)	0.41	0.50
Lumbar spine BMC (g/cm^2)	0.74 (0.08)	0.76 (0.09)	0.73 (0.05)	0.77 (0.09)	-0.02 (-0.03, 0.01)	0.28	0.50
**Parents**	**N = 16**	**N = 12**	**N = 16**	**N = 12**			
Body weight (kg)	75.0 (15.8)	64.6 (9.5)	74.8 (15.4)	64.4 (9.6)	0.0 (-0.7, 0.7)	0.71	0.00
BMI (kg/m^2)	26.5 (3.9)	23.8 (2.9)	26.5 (3.9)	23.7 (3.0)	0.1 (-0.1, 0.3)	0.85	0.17
**Dogs**	**N = 16**	**N = 12**	**N = 16**	**N = 12**			
Body weight (kg)	18.2 (10.1)	15.0 (7.9)	17.9 (10.0)	14.8 (8.3)	-0.1 (-0.2, 0.5)	0.63	0.17
Body condition score	2.9 (0.4)	3.1 (0.5)	3.0 (0.0)	3.1 (0.3)	0.1 (-0.1, 0.3)	0.38	0.25

Table 4. Child Health Related Quality of Life for intervention and control groups

	Baseline		Follow-up				
	Intervention Mean (SD)<br><br>N = 17	Control Mean (SD)<br><br>N = 13	Intervention Mean (SD)<br><br>N = 17	Control Mean (SD)<br><br>N = 13	Difference in change between intervention and control (95% CI)	p value	Effect size (Cohen's d)
**Self report**							
Physical health	82.1 (20.7)	88.7 (12.8)	86.2 (9.4)	87.7 (13.4)	5.1 (0.1, 10.1)	0.80	0.38
Psychosocial health	84.2 (11.3)	82.6 (15.3)	80.5 (13.8)	80.3 (18.6)	-1.4 (-5.7, 2.9)	0.78	0.12
Total score	83.5 (10.7)	84.7 (13.7)	82.5 (11.0)	82.9 (15.5)	0.9 (-2.9, 4.6)	0.88	0.01
**Proxy report**							
Physical health	84.2 (12.4)	90.6 (12.6)	84.7 (20.2)	87.5 (17.7)	3.6 (-4.7, 12.0)	0.74	0.16
Psychosocial health	79.2 (10.3)	80.9 (11.4)	83.2 (12.2)	81.8 (13.3)	3.1 (-1.5, 7.7)	0.58	0.25
Total score	81.0 (9.4)	84.3 (10.6)	83.7 (12.9)	83.8 (14.0)	3.3 (-2.3, 8.8)	0.84	0.22
